# Outbreak of equine herpesvirus 4 (EHV-4) in Denmark: tracing patient zero and viral characterization

**DOI:** 10.1186/s12917-024-04149-x

**Published:** 2024-07-03

**Authors:** Pia Ryt-Hansen, Victoria Kyhl Johansen, Marta Maria Cuicani, Lars Erik Larsen, Sanni Hansen

**Affiliations:** 1 Department of Veterinary and Animal Sciences, Faculty of Health and Medical Sciences, University of Copenhagen, Grønnegårdsvej 2, Frederiksberg C, DK-1870 Denmark; 2 Statens Serum Institut, Artillerivej 5, Copenhagen S, 2300 Denmark; 3 Department of Veterinary Clinical Sciences, Faculty of Health and Medical Sciences, University of Copenhagen, Taastrup, Denmark

**Keywords:** Equine herpesvirus 4, Outbreak, EHV-4, Patient zero, Full genome sequencing, Respiratory disease, Biosecurity

## Abstract

**Background:**

Equine herpesvirus 4 (EHV-4) causes respiratory disease in horses, and the virus is considered endemic in the global equine population. However, outbreaks can occur when several horses are gathered in relation to shows, competitions, breeding units and at hospitals. In the spring year 2022, an EHV-4 outbreak occurred at the Large Animal Teaching Hospital, University of Copenhagen, Denmark. Nine horses were tested EHV-4 positive during the outbreak, which lasted approx. seven weeks. In addition, a tenth horse “Eq10” tested EHV-4 positive almost three weeks after the last of the outbreak horses tested positive. Detailed clinical registrations were obtained from all ten horses as well as their location and movement during hospitalization. Nasal swabs were obtained throughout the outbreak and tested by qPCR for EHV-4. Additionally, pre- and post-infection sera were tested for the presence of EHV-4 antibodies. Selected samples were characterized by partial and full genome sequencing.

**Results:**

The most common clinical signs of the EHV-4 infected horses during this outbreak were pyrexia, nasal discharge, mandibular lymphadenopathy and increased lung sounds upon auscultation. Based on the locations of the horses, EHV-4 detection and antibody responses the most likely “patient zero” was identified as being “Eq1”. Partial genome sequencing revealed that Eq10 was infected by another wild type EHV-4 strain, suggesting that the hospital was able to eliminate the outbreak by testing and reinforcing biosecurity measures. The complete genome sequence of the outbreak strain was obtained and revealed a closer relation to Australian and Japanese EHV-4 strains rather than to other European EHV-4 strains, however, very limited sequence data are available from Europe.

**Conclusion:**

The study illustrated the transmission of EHV-4 within an equine facility/hospital and provided new insights into the viral shedding, antibody responses and clinical signs related to EHV-4 infections. Finally, sequencing proved a useful tool in understanding the transmission within the hospital, and in characterizing of the outbreak strain.

**Supplementary Information:**

The online version contains supplementary material available at 10.1186/s12917-024-04149-x.

## Background

In the family of *Herpesviridae*, nine equine herpesviruses (EHV-1-EHV-9) have been defined, with EHV-1, EHV-3, EHV-4, EHV-6, EHV-8 and EHV-9 belonging to the subfamily of *Alphaherpesvirinae* [1]. EHV-1 and EHV-4 are two important agents in relation to upper respiratory tract infections in horses [2]. The two viruses are closely related both genetically and antigenically with considerable level of immunological cross reactivity [3]. However, EHV-1 distinguishes from EHV-4 by frequent induction of abortions and neurological disease [2–5].

The genome of EHV-4 consists of linear double stranded DNA, approximately 145 kilo base pairs (kbp) in length. The genome contains 79 open reading frames (ORFs) [5] encoding 76 homologous genes, with three genes (ORF 64, 65 and 66) which are duplicated within the repeat regions [3].

EHV-4 infection can lead to respiratory disease [6] with clinical signs including lethargy, anorexia, pyrexia, mandibular lymphadenopathy, coughing and nasal discharge [4, 6, 7] with a duration between two and 14 days [4, 8]. Clinical disease is most often reported in foals and young horses [4, 6]. EHV-4 is transmitted by aerosols and by direct contact [7, 9]. Studies have reported a seroprevalence of > 80% in different geographical locations [4]. A key property of EHV-4 is its ability to establish latent infections. One study revealed that 56/70 horses, euthanized due to none-infectious causes, tested EHV-4 positive in the trigeminal ganglia [10]. Following the establishment of latency, EHV-4 can be periodically reactivated due to stressors such as transport, other disease, new environment etc., with recrudescence and shedding of EHV-4 as a result [2, 4, 7]. Several studies have performed whole genome sequencing [], whereas other studies solely focused on sequencing of ORF30 and ORF33 [11, 12], encoding a catalytic subunit of replicative DNA polymerase and an envelope glycoprotein, respectively. Whole genome sequencing analysis have shown 98.9–99.9% shared nucleotide identity among different Australian EHV-4 strains, corresponding to 179 single nucleotide polymorphisms (SNPs) [3]. Conversely, a German outbreak at a breeding stable revealed 99.9% identity corresponding to 76–98 SNPs [4].

The aims of this study were to characterize the genome of an EHV-4 outbreak strain and to describe the transmission and clinical disease related to an acute EHV-4 at a referral hospital.

## Results

### Real-time qPCR results of nasal swabs (Laboklin and VCM analyses)

**Fig. 1:**
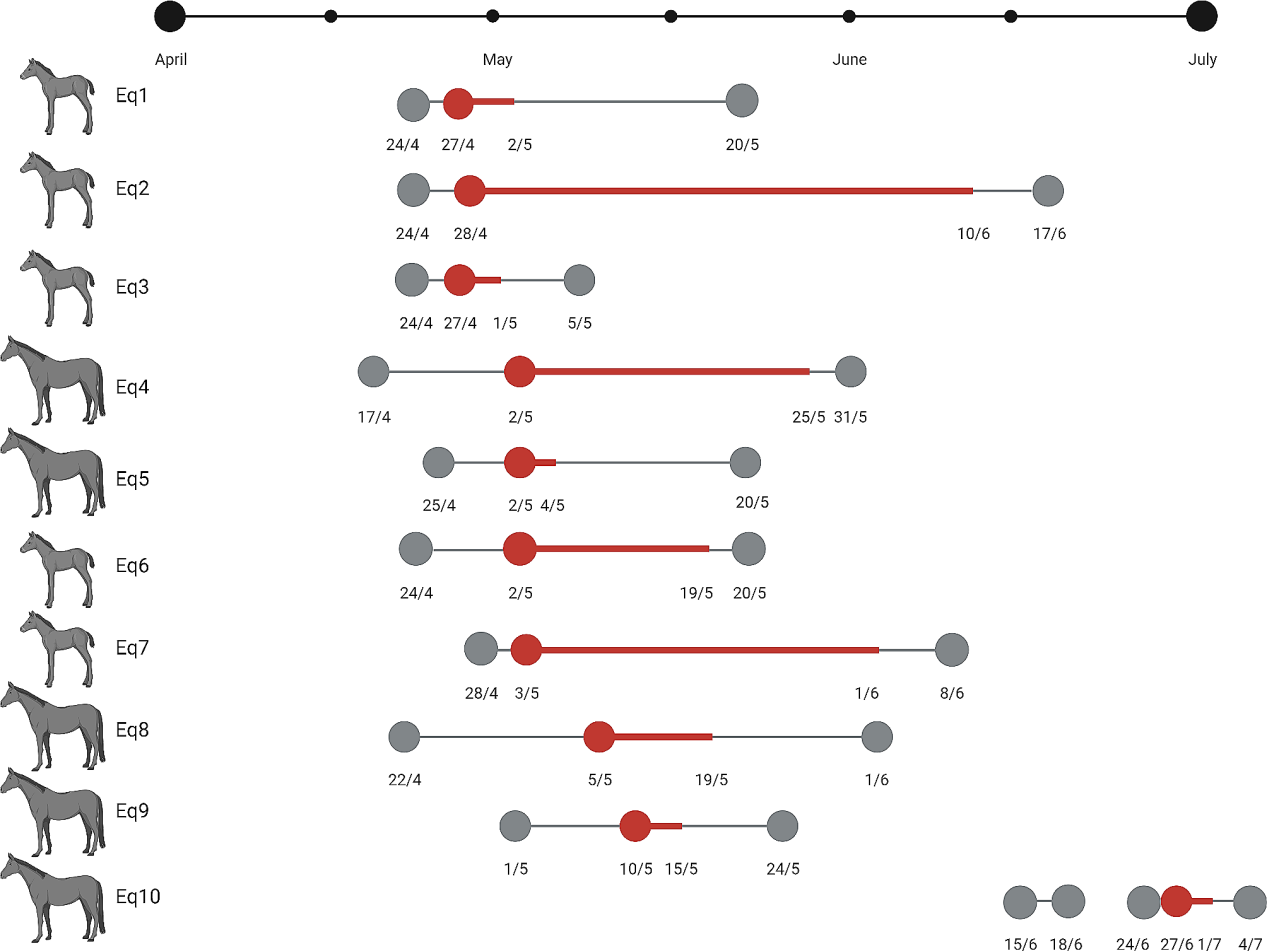
Timeline of the hospitalizations and EHV-4 detections of Eq1-Eq10 The figure illustrates the duration of hospitalization of each of the ten horses (grey) and period from when each horse had its first nasal swab collected that tested EHV-4 positive until the last collected nasal swab tested positive (red). The black horizontal line represents the timeline of the study from April-July 2022. The specific dates of the collection of the nasal swabs are indicated underneath the timelines of each horse. The qPCR test results are both derived from the analyses performed at Laboklin and VCM, and negative PCR results within the “detection period” (red) are not included. The horses are illustrated as either foals ≤ 1 year old (foal symbol) or adult horses > 1 year old (horse symbol)

Initially, the first three horses showing clinical signs of respiratory disease (Eq1, Eq2 and Eq3) all tested positive for EHV-4 (Fig. 1), EHV-5 and Streptococcus equi subspecies zooepidemicus. Additional four horses tested EHV-4 positive after 4–5 days (Eq4, Eq5, Eq6 and Eq7), and the last two horses (Eq8 and Eq9), tested positive eight and 13 days after the first horse tested EHV-4 positive, respectively.

Eq10 was referred to the hospital for the first time, while the last EHV-4 positive horses (Eq2) was still isolated in the quarantine. However, Eq10 was re-admitted to the hospital 17 days after Eq2 tested EHV-4 positive for the last time.

Three horses did not have consecutive positive EHV-4 tests during the “infection period” (Additional File 1). All serum samples tested negative for the presence of EHV-4 by qPCR, except for Eq2, that had one blood sample obtained the 5th of May 2022, with a Ct value of 34.49. A detailed overview of the real-time qPCR results from both laboratories are shown in Additional File 1.

### Pre- and post-infection antibody responses

**Fig. 2:**
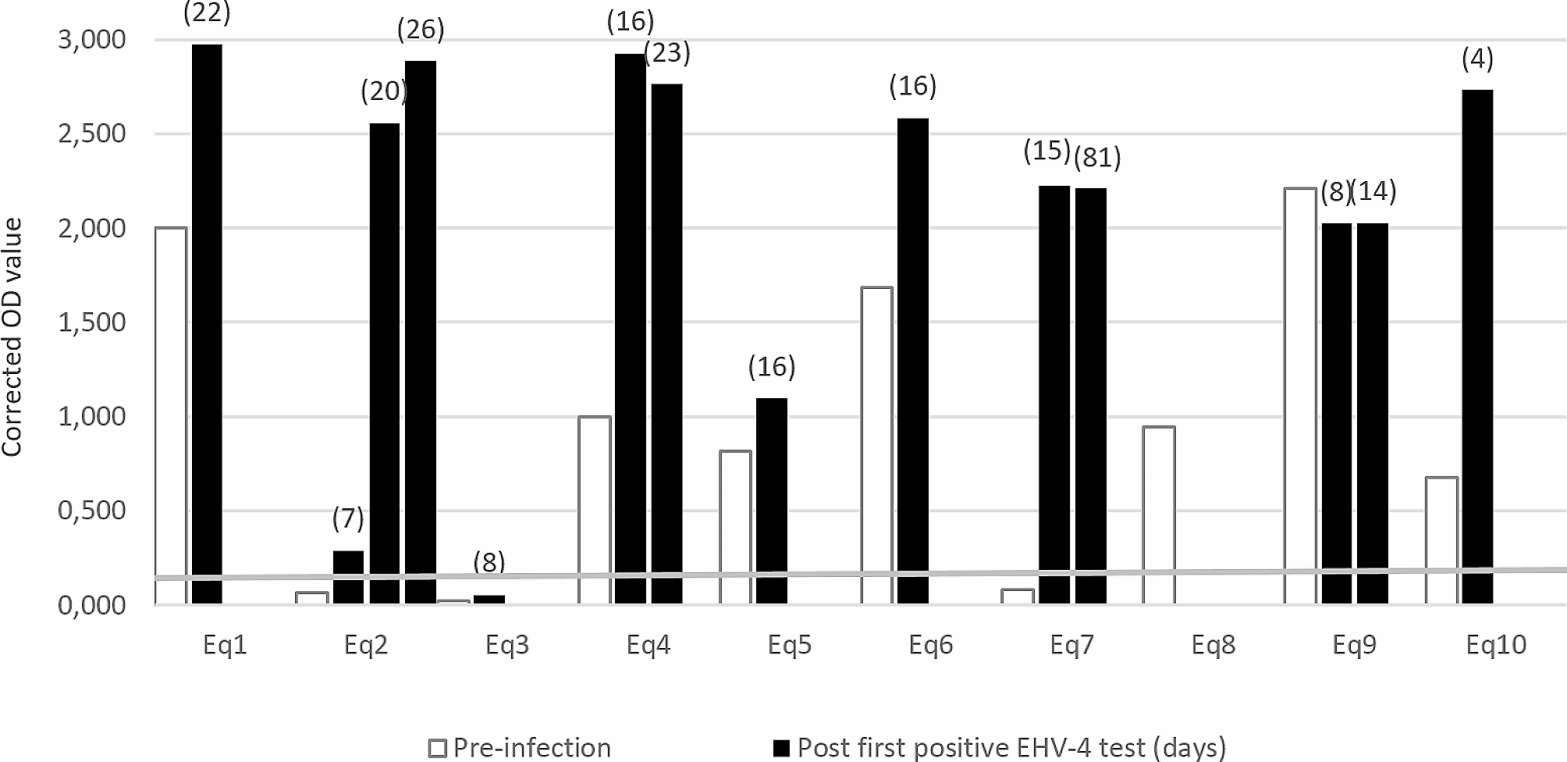
EHV-4 antibody ELISA results of the sera obtained from the ten horses pre- and post-infection presented as corrected OD-values For the post-infection sera the number of days after the initial EHV-4 positive real-time qPCR test results are presented in the parenthesis above the columns. Antibody negative samples are marked with bold letters. The pre-infection sera were obtained at arrival, but for Eq8 and Eq10 additional pre-infection sera were also analyzed (Eq8 = 1.29 and Eq10 = 0,707). In addition, Eq2, Eq4, Eq7 and Eq9 had several post-infection sera analyzed. The grey horizontal line indicates the 0.2 cut-off for a positive ELISA result

The ELISA results of the pre- and post-infection sera revealed that only three of the ten horses were naïve to EHV-4 antibodies at the time of arrival at the hospital (Eq2, Eq3 and Eq7) (Fig. 2). These were all foals being between ten months to one year of age (Additional File 2). Two of these foals subsequently tested positive in the ELISA following EHV-4 infection, whereas the last foal remained negative (Eq3). However, the post-infection sera of this foal was obtained only eight days after the initial EHV-4 positive test result. The remaining seven horses were seropositive already upon arrival. In total, seven horses showed an increase in EHV-4 antibodies following infection. Seven out of the ten horses from the outbreak had an OD value < 1.0 at arrival at the hospital, whereas eight of the horses had an OD value > 1.0 post infection, with seven of them being > 2.0.

### Clinical signs

Clinical signs related to the ten EHV-4 infected horses during the outbreak are summarized in Table 1 and included pyrexia, mandibular lymphadenopathy, nasal discharge, ocular discharge, coughing, increased lung sounds and anorexia. Nasal discharge was the most common clinical sign along with pyrexia and increased lung sounds. Detailed clinical registrations for each horse are listed in Additional File 3. Based on clinical signs, the ten infected horses could be divided into three groups. One group included three horses (Eq4, Eq5 and Eq9) more than ten years old that showed no clinical signs of respiratory disease, or had one day with nasal discharge. The second group consisted of horses below one year of age that were moderately affected characterized by being subfebrile and with mandibular lymphadenopathy, nasal discharge, and increased lung sounds (Eq1, Eq2 and Eq6). Eq1 and Eq2 also had two and one day of mild fever, respectively and Eq2 had several days with coughing registered. The third group included three horses between 11 months to two years of age (Eq3, Eq7, Eq8), and one 7-year-old horse (Eq10) being severely affected with pyrexia for three-five days, nasal discharge and increased lung sounds. None of the severely affected horses had mandibular lymphadenopathy. The reason for admission to the Large Animal Teaching Hospital and other non EHV-4 related clinical signs observed during their hospitalization are listed in Additional File 2.

**Table 1:** Summary of the clinical signs of the ten EHV-4 infected horses

Clinical sign:	Pyrexia	Mandibular lymphadenopathy	Nasal discharge	Coughing	Increased lung sounds	Anorexia	Ocular discharge
Prevalence	6/10	5/10	9/10	4/10	6/10	2/10	5/10
Average no. of days	3.33	13.6	4.9	2	3	4.5	1.8

The prevalence indicate how many of the ten horses presented with the specific clinical sign

### Tracing movements of the horses

**Fig. 3:**
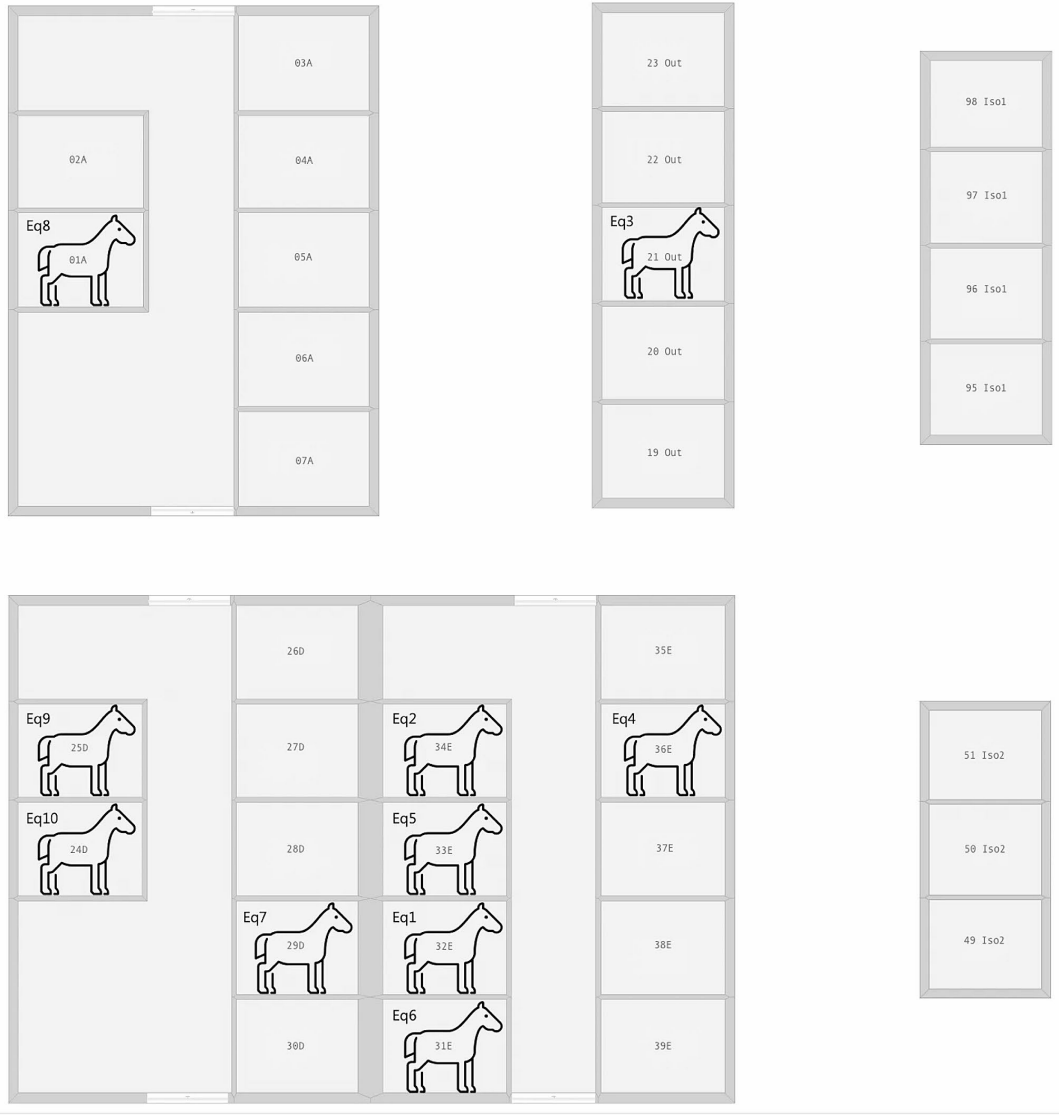
Overview of different stables of the Large Animal Teaching Hospital and the location of the horses at their initial EHV-4 positive test The figure represents a simplified overview of the different stables at the Large Animal Teaching Hospital, the true distances are not illustrated but white areas indicate that the stables are in separate buildings. Additional horses were housed in the same stables of the hospital during the outbreak, but only EHV-4 positive horses are included in this figure. “Iso1” and “Iso2” indicates the isolation units and “Out” indicates outdoor stables with open boxes to the outside. The location of Eq10 at its initial hospitalization is also included even though it was not tested for EHV-4 at this time

Eq1 stabled in box 32 E, and Eq3 stabled in the outside box, were the first two horses to test positive for EHV-4 (Fig. 3). The horses were transferred to the isolation unit 1 following their first EHV-4 positive test, and remained there until they tested negative. Eq2, Eq4 and Eq5 were all housed in stable E, and were later moved to isolation units 1 or 2. The horses remained individual isolated until being discharged from the hospital. Stable E was closed for intake of patients during most of the outbreak and acted as quarantine for other close-contact horses that had been in contact with the first positive confirmed infected horse, and later in the outbreak period, it became isolation unit due to lack of space in the isolation unit. Eq6 remained in stable E during its entire hospitalization. Eq7 and Eq9 was initially housed in stable D, but after testing positive for EHV-4, they were moved to the isolation unit 1 and 2, respectively. Eq8 was initially in the intensive care unit (stable A) and was moved to the isolation unit 2 when it developed clinical signs of disease. The remaining horses in stable A tested negative for EHV-4. Eq10 was initially hospitalized for minor surgery of skin tumors, and was stabled in stable D during its hospitalization. It was installed in a box where no EHV-4 positive horse had been stabled during the outbreak and one months after the last EHV-4 positive horses had left this stable. At the time of surgery of Eq10, all but one horse from the outbreak had been discharged, and the last EHV-4 infected horse was stabled in the isolation unit 1. Eq10 was discharged from the hospital three days following the surgery. When the horse was re-admitted to the hospital six days later, showing clinical signs of respiratory disease, it was stabled in isolation unit 1 upon arrival. Biosecurity was tightened during the outbreak to limit contamination and transmission. The horses were stabled in green, yellow and red zones, with different personnel for each section and in the yellow and red zone, the personnel had to wear protective clothing, that were changed between each individual horses.

Until the first EHV-4 positive test results, the hospitalized horses were removed from their boxes to be examined and exercised, thereby introducing possible transmission routes. However, during the outbreak, infected or close-contact horses were only taken to an examination room in urgent circumstances and with proper disinfection and rest time after. Within the stables A, E and D the horses could have direct and indirect contact with the horses in neighboring boxes despite that the walls of the box being were around 2.5 m high, and with wooden planks separating the boxes. The outdoor boxes also had shared airspace, but with no possibility for indirect or direct contact. The boxes of the isolation units were completely separated from each other, so that the horses could not have any contact, although they still shared airspace.

### Virus isolation in cells

Following four passages in the Equine dermal cells, a viral isolate was successfully obtained from Eq7 with a Ct value of 12.4 in the real-time qPCR carried out at VCM.

### Analyses of the complete EHV-4 genome of the Danish outbreak strain

**Fig. 4:**
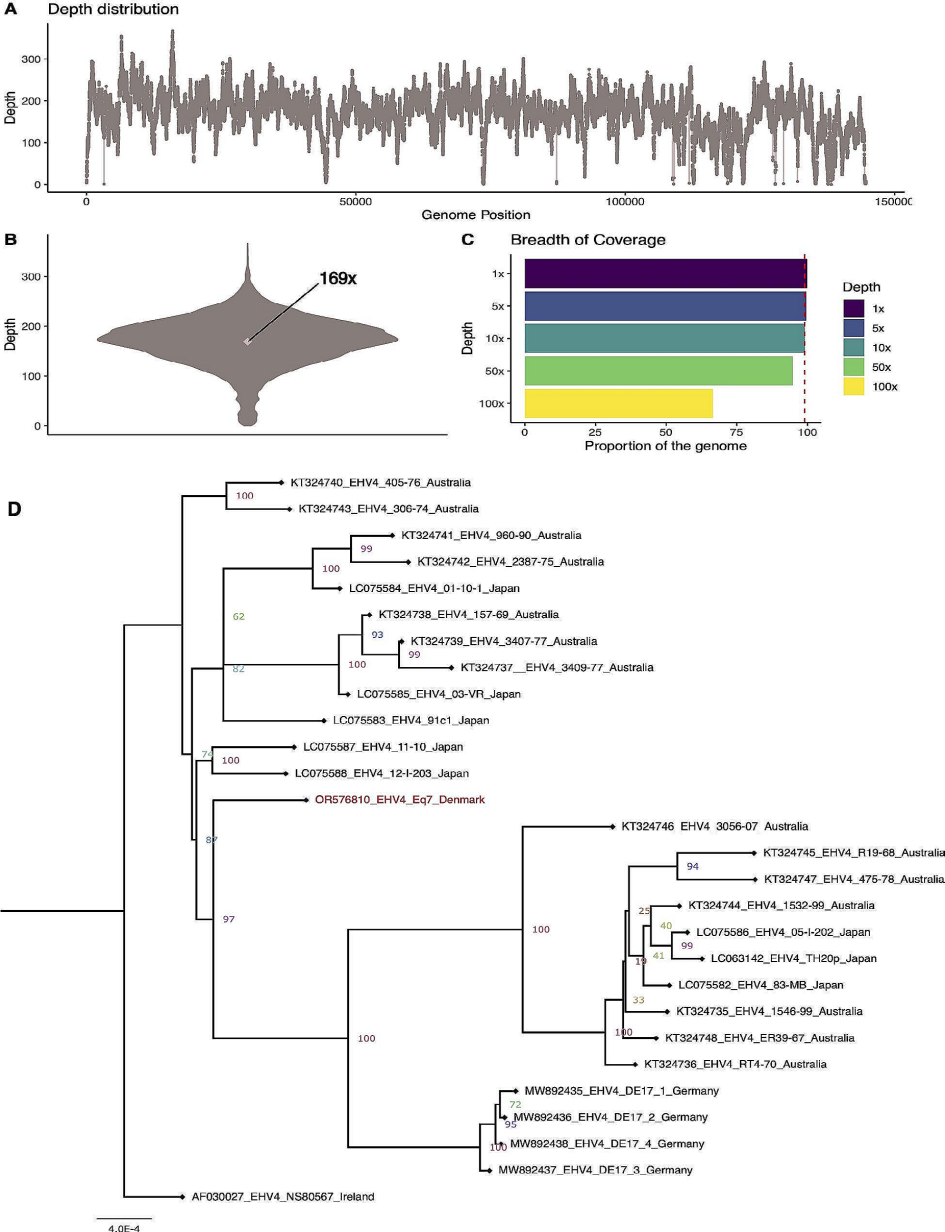
Genomic characterization of Eq7 (**A**) Depth distribution across the genome. (**B**) Violin plot showing the average depth of coverage. (**C**) Plot showing the breadth of coverage at 1x, 5x, 10x, 50x and 100x. The red dotted line represents a threshold of 99%. (**D**) Maximum likelihood tree based on the whole genome sequencing data from the sample Eq7 and the 27 reference sequences available from NCBI Genbank. The bootstrap support is shown at the base of each node. The accession number of each reference sequence is indicated in the taxon

We successfully obtained a complete genome from the viral isolate of Eq7 accession number: OR576810 in NCBI GenBank. The raw data contained ∼ 23.000 paired end reads which after filtering and mapping remained ∼ 4700 reads with a proportion of endogenous content equal to 20.6%. After mapping and filtering the average coverage of the sample was approx. 169x (Fig. 4A and B) with 99.87% of the genome that was covered at least 1x, 99.55% was covered 5x, 99.03% was covered 10x and 94.85% was covered 50x (Fig. 4C). Low coverage regions (below 25x) were specifically identified in areas associated to repeated regions in the Irish reference genome and were more prevalent towards the end of the genome (Fig. 4A). The consensus genome for Eq7 was generated and used together with one Irish, four German, eight Japanese and 14 Australian whole genome sequences to build a full genome phylogeny. The Maximum Likelihood tree was highly supported and revealed the Danish EHV-4 genome as closest to a cluster of sequences from Japan and Australia (Fig. 4D). The number of nucleotide dissimilarity between our sample (OR576810_EHV4_Eq7_Denmark) and the rest of the full genome sequences in the dataset ranged from 348 to 782 bases with the highest sequence identity to Equid alphaherpesvirus 4 isolate 405 − 76 from Australia (NCBI Genbank accession number: KT324740).

### Analyses of the partial ORF30 sequences

**Fig. 5:**
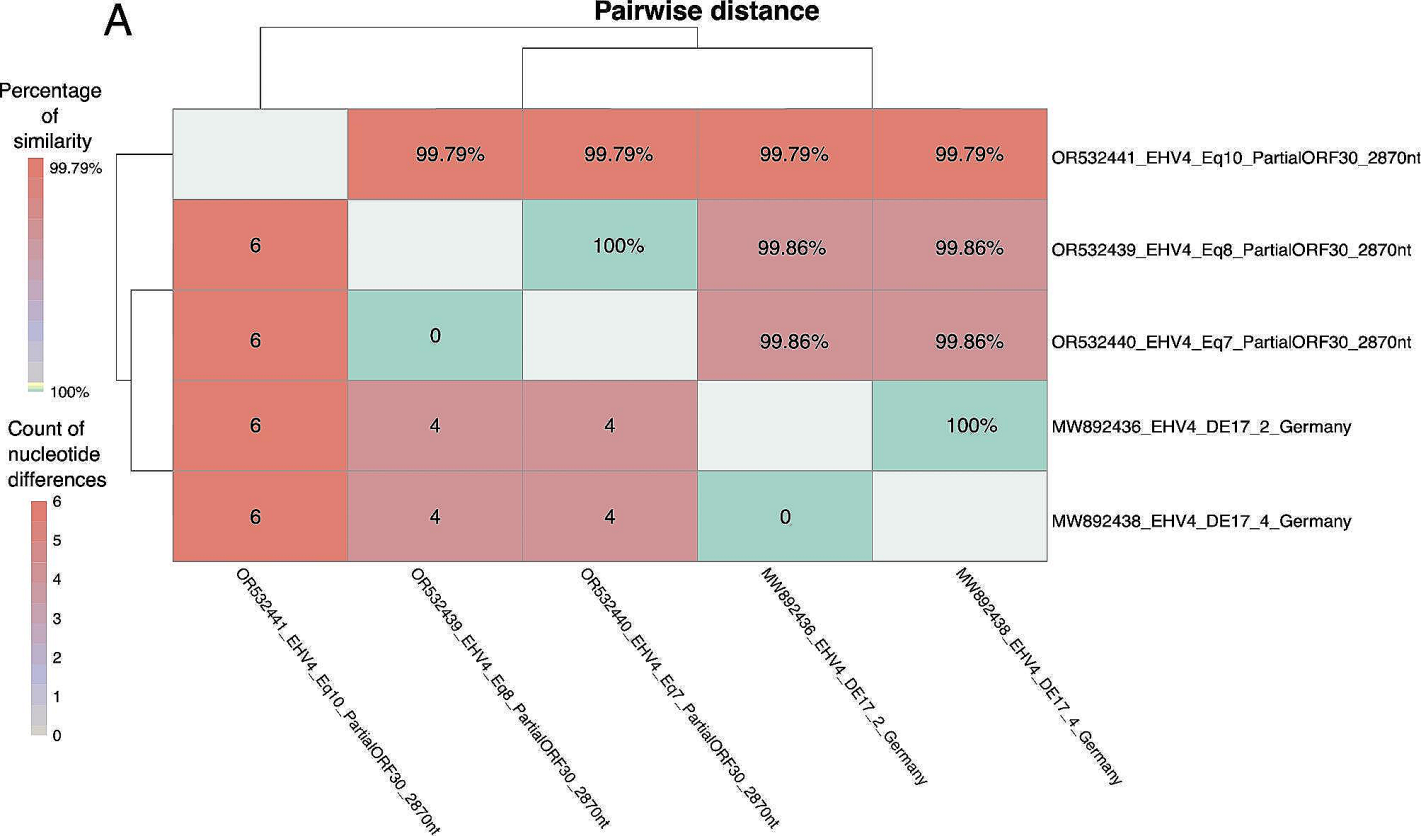
Characterization of the partial ORF30 sequences of Eq7, Eq8 and Eq10 Pairwise distance plot of the three partial ORF30 EHV-4 sequences obtained from the three Danish horses (Eq7, Eq8 and Eq10) and two selected German reference sequences with the NCBI Genbank accession numbers: MW892436 and MW892438. The bottom section of the plot shows the count of nucleotide differences while the upper part shows the percentage of identity among the sequences

In total, 2870 nts of the ORF30 were successfully obtained from three of the EHV-4 infected horses including Eq7 (accession number OR532440), Eq8 (accession number OR532439) and Eq10 (accession number OR532441). Subsequently, a pairwise comparison was performed including the three partial ORF30 sequences. The results of this sequence comparison revealed that the ORF30 of two of the horses Eq7 and Eq8 were 100% identical, whereas the sequence of Eq10 showed six nucleotide differences compared to Eq7 and Eq8. The partial ORF30 sequences were also compared to two German reference sequences obtained from another outbreak. These two German sequences were also 100% identical to each other, while only four nucleotide differences were observed to Eq7 and Eq8 and six nucleotide differences were found to Eq10 (Fig. 5).

## Discussion and conclusions

Although EHV-4 is widespread and enzootic circulating in horses globally, there is very few published studies on outbreaks linking viral transmission and excretion with clinical signs. In the present report, we describe an outbreak of EHV-4 in a large university hospital. The data collected are unique in the sense that both nasal swab and blood samples were available from all horses also before the first horse tested positive, detailed clinical examinations were performed on a daily basis and the hospital recorded the housing and movement of horses during the full course of the outbreak.

On average, the EHV-4 infected foals and horses included in this study tested PCR positive on nasal swabs for viral DNA for approximately 17 days. This is in accordance with recent studies showing that 75% of EHV-4 infected foals shed viral DNA in nasal secretion for two to four weeks, measured by PCR in nasal swabs [4, 8]. Results from the present study found that only one serum sample tested weakly positive for EHV-4 virus, which is in accordance with a study showing that EHV-4 could only be detected in low concentration in peripheral blood leukocytes in the early state of infection compared to the amount of virus detected in nasal swabs [10].

A previous study showed that detection of EHV-4 mRNA as a measure of active replication correlated well with both clinical signs and detection of high levels of EHV-4 DNA and lasted for approximately 7 days [10], and EHV-4 could be isolated in cell culture for up to three days after experimental infection [13]. None of these methods can precisely predict the period where infected horses can transmit the virus on to other horses as the PCR tests can pick of reminiscence of non-infectious viral RNA and the detection limit of the cell culture isolations is probably above the infectious dose. Furthermore, the infectious dose will depend on a variety of individual host -and environmental factors. Nevertheless, based on findings from the present study, the epidemiological information and results of previous studies it is reasonable to estimate that on average an infected horse may transmit the virus on to other susceptible horses 2–9 days after infection, but with significant individual differences. As observed in previous studies, the EHV-4 infected horses of the Danish outbreak presented with clinical signs such as anorexia, pyrexia, mandibular lymphadenopathy, coughing and nasal discharge [4, 6, 7]. The majority of the ten horses showed nasal discharge, lymphadenopathy and increased lung sounds and 8/10 horses were either subfebrile or had pyrexia during their infection. An additional observation in this Danish outbreak, was that 50% of the horses also had ocular discharge, which was also reported by an American study [13].

The four most severely affected horses were between 11 months and two years of age with the exception of one horse being 7 years old. All had a low EHV-4 pre-infection antibody levels. On the contrary, the horses classified as subclinical to mildly affected were all above ten years old. Five of the ten infected horses in the Danish outbreak were younger than one year of age which in in accordance with previous studies finding that foals less than one year are more often infected [4, 8, 14]. This indicate that both age and the immune status at the time of infection plays an important role in the severity of disease. The three horses that were EHV-4 naïve upon arrival at the hospital were all ≤ 1 year old, thereby making it likely that they did not encounter EHV-4 before. Two of the three antibody negative horses seroconverted after the EHV-4 infection, whereas the third horse remained seronegative, probably because the last available blood sample was obtained only eight days following the first EHV-4 positive test, which may be before a detectable antibody response can be detected. An increase in OD-value was observed in five out of six horses, from which a post-infection serum sample was obtained. The majority of the post-infection sera was obtained two-three weeks after the first EHV-4 positive nasal swab. Interestingly an 81 days post-infection serum sample from Eq7 was obtained with no significant decrease in the OD-value compared to the sample taken 66 days earlier.

Based on the viral detections, antibody responses, clinical registrations, stable locations and horse movement it was possible to identity Eq1 as a potential “patient zero” of the outbreak. Only two horses, Eq1 and Eq9, had high levels of EHV-4 antibody titer at arrival to the hospital, indicating that they could have had an active EHV-4 infection or re-activation of latent EHV-4 virus due to stress induced by recent disease, transportation and/or hospitalization. Re-activation is known to cause a boost of prior EHV-4 antibody response resulting in the high antibody levels observed [15]. As Eq9 was hospitalized several days after the first three confirmed EHV-4 cases this horse was ruled out as patient zero.

The first horses to present with EHV-4 positive nasal swabs and clinical signs of disease were Eq1, Eq2 and Eq3 Eq2 and Eq3 were both negative for EHV-4 antibodies at arrival to the hospital, however, a rise in antibodies post infection is not measurable until 8–10 days after infection [14]. Eq2 and/or Eq3 could therefore possibly have been infected shortly before being hospitalized and can thereby not be entirely ruled out as patient zero. Nevertheless, the fact that Eq1 were among the first to show clinical signs combined with its antibody positive status point at this horse being patient zero. Noteworthy is also that Eq6 also showed clinical signs of disease at an early point and had a relatively high level of antibodies at arrival at the hospital, however, this horse was not tested until a week after the three first confirmed EHV-4 cases. Interestingly, Eq6 originated from the same premise as Eq1, and the two horses were transported and admitted to the hospital together.

Five out of ten horses were stabled in stable E, the location of two of the first EHV-4 positive horses, possible creating a high-risk environment for transmission both by direct contact trough aerosols [16] or indirect contact such as personal, use of the same equipment, examination rooms and feeding trucks. The persistence of EHV-4 in the environment has not previously been reported, but it has been shown that EHV-1 can persist for up to 48 h [17] and for three weeks in water [18].

Eq10 was admitted to the hospitalized two months after the beginning of the EHV-4 outbreak at a time where only one EHV-4 infected horse was present in the isolation unit. Results of the partial ORF30 sequencing of the strain from Eq7 and Eq8 only showed 99.79% sequence identity to that of Eq10, equal to the genetic difference between German, Australian and Japanese sequences (Pavulraj et al. 2021) indicating that Eq10 was infected with another EHV-4 strain than the one related to the outbreak (Fig. 5). This in turn indicate that the internal biosecurity measures at the hospital were able to eliminate the outbreak virus. The presence of different EHV-4 strains within a short period indicate that EHV-4 is enzootic and stress the importance of biosecurity in equine hospital facilities where several horses are housed together, have co-morbidities and high stressors that can re-activate a latent EHV-4 infection. In the present study, the three initially infected horses were also positive for co-infections with EHV-5 and Streptococcus Equi subsp. Zooepidemicus. Both pathogens are known commensals, but can potentially enhance disease [6, 9, 19]. Interestingly, the three horses with the co-infection belonged to the moderately or severely clinically affected groups. A recent study by Pusterla et al. found that 20% of EHV-4 infected horses were co-infected with one or multiple of the following pathogens: Equine Influenza virus, Streptococcus Equi subsp. Equi and Equine Rhinitis virus [6] but the horses of that study were not tested for Streptococcus Equi subsp. Zooepidemicus or EHV-5.

Interestingly, the full genome sequence of the EHV-4 virus isolated from Eq7, revealed a higher level of sequence identity to EHV-4 strains from Australia and Japan, than to other European EHV-4 sequences available from Germany (Fig. 4). Since very few full genome EHV-4 sequences are available for comparison these results should be interpreted with caution. More global EHV-4 sequences are needed to be able to analyze local clustering and exchanges of EHV-4 viruses.

## Methods

### Study design

Included in the study were nine horses (mean age of five years), that were all part of an EHV-4 outbreak at a referral hospital in Denmark. The exact age and additional information are available in Additional File 2. Inclusion criteria was a minimum of one EHV-4 positive nasal swab analyzed by the commercial laboratory Laboklin, Germany. Horses hospitalized during the outbreak and tested negative for EHV-4 were not included in the study.

The “outbreak period” defined as the period from the first horse tested EHV-4 positive (27th of April) until the last EHV-4 positive horse was discharged after testing negative lasted for a total of seven weeks (17th of June 2022) with the last test positive horse testing positive June 10th 2022. The given dates indicate the collection day of the nasal swab that later tested positive in the laboratory. Horses displaying clinical signs compatible with EHV-4, and close-contacts of infected horses, were all tested by nasal swabs send for analysis at a commercial laboratory. Isolation of EHV-4 positive horses and close-contact horses was initiated following the American Association of Equine Practitioners (AAEP) Biosecurity Guidelines [20]. In addition, all EHV-4 positive horses were tested continuously until they tested negative. A selection of these samples was also tested at the Section of Veterinary Clinical Microbiology at the University of Copenhagen (VCM). Clinical files, stable/box locations and paraclinical test results were included from the infected horses.

An additional horse, Eq10, initially hospitalized from the 15th -18th of June 2022 and re-admitted to the isolations facilities on June 24th 2022 due to pyrexia was also included into the study, as it tested positive for EHV-4 on the 27th of June 2022. The clinical file and samples similar to the once described above was included for this horse. Additional file 4 describe the complete sample list.

### Nasal swab for PCR

Nasal swabs for analyses at a commercial laboratory were obtained by the use of 15 cm long dry swabs (Kruuse, Denmark), passed as far as possible into the nasopharynx by the ventral meatus and rotated for 15–30 s. The swabs were inserted into an empty sterile container and kept in a refrigerator until being shipped and analyzed by the laboratory. The first samples were tested for a number of respiratory pathogens including: EHV-1, EHV-5, Streptococcus Equi subsp. Zooepidemicus and Streptococcus Equi subsp. Equi, Coronavirus and Influenza A, whereas the last samples were tested solely for the presence of EHV-4 by real time PCR. The time span between sampling and test results was approximately 3 working days. In addition to the nasal swabs obtained and analyzed by the commercial laboratory during the outbreak, additional nasal swabs (Medical Wire, Corsham, UK) were collected from the EHV-4 positive horses, for analysis at VCM. The swabs were subsequently immersed in Sigma Virocult Media (Medical Wire) and stored at minimum − 20 °C until further analysis. In total, 28 nasal swabs were tested at VCM, which together with the results of the nasal swabs tested at Laboklin (*n* = 38) equaled 66 qPCR results on EHV-4 detection from the ten horses.

### Clinical findings

Each horse was clinically examined at least once daily. The following data was extracted from the files: rectal temperature, palpation of the mandibular lymphnodes (size and painfulness), the presence or not of nasal discharge (colour and viscocity), the presence of cough (yes/no), lung auscultation (normal/abnormal) and the presence or not of ocular discharge.

### Serum sample collection, storage, and handling

Serum samples were routinely drawn from all horses at admittance and on indication during the stay. Samples were stored at -20 C. All possible serum samples obtained from the ten outbreak horses were included (*n* = 26) with the aim of obtaining one pre-infection and at least one post-infection serum sample from each horse.

### Extraction of DNA

The DNA extraction of the 28 nasal swab samples analyzed at VCM laboratory was performed using the QIAamp DNA Mini Kit (QIAGEN, Hilden, Germany) automated on the QIAcube connect (QIAGEN), according to manufacturer’s protocol [16]. The DNA of the serum samples and the viral isolate were extracted manually using the same extraction kit.

### Real time qPCR

The extracted DNA was subsequently used in a previously published real-time qPCR targeting the glycoprotein B of EHV-4 [8]. In brief, 20 𝜇L of reaction mix (Sensifast No-Rox (2x), primers (EHV4-F: CGCAGAGGATGGAGACTTTTACA and EHV4-R: CATGACCGTGGGGGTTCAA) and probe (EHV4-P: FAM-CTGCCCGCCGCCTACTGGATC-TAM)) was mixed with 5 𝜇L of DNA and analyzed on the Rotor-gene Q machine (QIAGEN) using the following program: 95 °C for 2 min, 40 × (95 °C for 3 s and 60 °C for 60 s (acquire on green channel)) and 60 °C for 60 s. The raw data was analyzed using the following settings: cycle threshold (Ct) 0.02, ignore the first cycle, using dynamic tube, slope correct, and an outlier removal of 10%. A positive and a negative control were included in all runs, and all samples were analyzed in duplicates.

### ELISA

The serum samples obtained were tested at the VCM laboratory for the presence of EHV-4 antibodies using a commercial ELISA [17] able to differentiate between EHV-1 and EHV-4 antibodies. As mentioned, the samples were selected to obtain minimum one pre- and one post-infection sample, with the post infection sample being as far in time from the first EHV-4 positive test as possible. The commercial kit was the Svanovir^®^ EHV1/EHV4-Ab kit (Svanova, Uppsala, Sweden), which is an indirect ELISA based on type-specific recombinant glycoprotein G fusion protein. The preparation of reagents and the ELISA procedure were performed according to the instruction manual [17]. A corrected OD value of > 0.2 was regarded as positive, whereas a corrected OD value < 0.1 was regarded as negative and a corrected OD value of 0.1–0.2 were considered as doubtful.

### Virus isolation in cells

Equine Dermal cells, NBL-6 (ATCC, Denmark) was cultivated using MEM (Gibco, Termofisher Scientific, Roskilde, Denmark), Non-Essential Amino Acids (Merck, Darmstadt, Germany), Na-Pyruvate 100mM (Gibco, Termofisher Scientific), Penicillin-Streptomycin-Neomycin (PSN) Antibiotic Mixture (Termofisher Scientific) and 10% Fetal Calf Sera. At 100% confluence, the cells were inoculated with 200𝜇L nasal swab sample of Eq7 that was first sterile filtrated using 0.45𝜇m Minisart NML Plus Surfactant-free Cellulose Acetate Syringe Filters (Sartorius, Göttingen, Germany). The cells and the supernatant were passaged using 0.05% Trypsin-EDTA (Gibco, Termofisher Scientific) four times adding 1/5 new cells in each passage in a larger cell flasks. At each passage 200𝜇L of cells were harvested and subjected to the DNA extraction and the real-time qPCR described above in order to determine if the viral isolation had been successful.

### Next-generation sequencing (NGS)

The supernatant and the cells of the fourth passage were harvested and centrifuged at 3000 RPM for 15 min to remove cellular debris. Thereafter the supernatant was subjected to ultracentrifugation at 25.000RPM for one hour at 4^0^C. The pellet containing the virions were then disrupted using 200μL PBS, and the DNA extracted manually using the DNA Purification protocol “Blood or Body Fluid“ of the QIAamp DNA mini kit (QIAGEN). The extracted DNA was used as input for the Nextera XT library prep protocol, and the samples were sequenced using the Illumina MiSeq platform (Statens Serum Institut, Copenhagen S, Denmark).

### Analysis of the NGS data

Illumina raw reads from the isolate of Eq7 were filtered using fastp v0.20.1 [18] to filter out low complexity reads and with quality below 25. The filtered reads were then aligned to the German reference genome (MW892436) using bwa v0.7.17 [21, 22] and potential duplicates were identified and removed using picard MarkDuplicates v2.27.2 (http://broadinstitute.github.io/picard). Samtools v1.16.1 [23] was then used to sort and index the bam file and to determine the read coverage along the whole genome. The consensus genome was generated using bcftools mpileup and bcftools call v1.14 [23]. Genomic position with read coverage below 5x were assigned as missing site “N”.

The consensus sequence was concatenated with the 27 full reference genomes retrieved from NCBI from Australia, New Zealand, Germany, Ireland and Japan (NCBI Genbank accession numbers: AF030027, KT324741, KT324740, KT324743, KT324746, KT324745, KT324744, KT324748, KT324736, KT324738, KT324739, KT324747, KT324742, KT324735, KT324737, MW892435, MW892436, MW892437, MW892438, LC075586, LC063142, LC075587, LC075582, LC075585, LC075584, LC075588 and LC075583).

The sequences were aligned using mafft v7.486 [24] and trimAl v1.4 [25] with a gap threshold of 95% and a conservation percentage of minimum 60% (-gt 0.95 -cons 60) was used to remove gaps related to highly repetitive regions. A pairwise comparison and a maximum likelihood phylogenetic tree of the full genome data was generated with IQ-TREE v2.1.2 [26] with the option -m TEST to calculate the best evolutionary model for the alignment using ModelFinder [27] and 1000 bootstrap replicates.

### PCR amplification of ORF30

For other EHV-4 samples, it was not possible to obtain an isolate, and therefore conventional PCR amplification of ORF30 was performed. Four primer-pairs previously published for ORF30 were used [4], along with the Accuprime Taq High Fidelity kit (Termo Fisher Scientific, Denmark). Four different PCR programs were applied for each primer pair (see Additional File 5). The resulting PCR products were visualized using the Invitrogen E-gel 1% SYBR Safe agarose gel, Termo Fisher Scientific and purified using the High Pure PCR Product Purification Kit, Roche, Basel, Switzerland. The PCR products were used as input for the Nextera XT library prep protocol, and the samples were sequenced using the Illumina MiSeq (Statens Serum Institut, Copenhagen S).

### Analysis of the ORF30 sequencing data

The data from the next-generation sequencing (NGS) was analyzed using the CLC genomics workbench version 22.0.2 (QIAGEN). Fastq files were imported, and all of the reads were paired and trimmed with a quality limit of 0.2 and a maximum number of ambiguities of two. The trimmed reads were then mapped to a full-length reference genome of a recent German EHV-4 strain (accession number: MW892438). A consensus sequence of ORF30 was extracted and subsequently aligned using the MUSCLE alignment tool [28] and to the partial ORF30 derived from all EHV-4 sequences available at NCBI GenBank described above (*n* = 27). Following the alignment, a “pairwise comparison” was created to examine nucleotide differences between Eq7, Eq8 and Eq10 and two German reference sequences (accession numbers: MW892436 and MW892438) derived from a single outbreak.

### Electronic supplementary material

Below is the link to the electronic supplementary material.

Supplementary Material 1

Supplementary Material 2

Supplementary Material 3

Supplementary Material 4

Supplementary Material 5

## Data Availability

All data generated or analyzed during this study are included in the article and its supplementary files.
