# Supplementary material for: Outbreak of equine herpesvirus 4 (EHV-4) in Denmark: tracing patient zero and viral characterization
Source: BMC Vet Res. 2024 Jul 3;20:287. doi: 10.1186/s12917-024-04149-x (PMC11221098; doi:10.1186/s12917-024-04149-x)
Supplement: Supplementary file 1 — Supplementary Material 1 [file 12917_2024_4149_MOESM1_ESM.docx]

|  | **Eq1** | **Eq2** | **Eq3** | **Eq4** | **Eq5** | **Eq6** | **Eq7** | **Eq8** | **Eq9** | **Eq10** |
| --- | --- | --- | --- | --- | --- | --- | --- | --- | --- | --- |
| 27-apr | LK+ |  | LK+ |  |  |  |  |  |  |  |
| 28-apr |  | LK+ |  |  |  |  |  |  |  |  |
| 29-apr |  |  |  |  |  |  |  |  |  |  |
| 30-apr |  |  |  |  |  |  |  |  |  |  |
| 01-maj |  |  |  |  |  |  |  |  |  |  |
| 02-maj | LK+ | LK+ | LK- | LK+ | LK+ | LK+ |  | LK- |  |  |
| 03-maj |  |  |  |  |  |  | LK+ |  |  |  |
| 04-maj |  |  |  |  |  |  |  |  |  |  |
| 05-maj | NS- | NS (31.05) and 34.5^ | NS- | NS (34.6) | NS- | NS (31.2) |  | LK+ / NS (24.1) |  |  |
| 06-maj |  |  |  |  |  |  |  |  |  |  |
| 07-maj |  |  |  |  |  |  |  | NS (20,01) |  |  |
| 08-maj |  |  |  |  |  |  | NS (17.24) |  |  |  |
| 09-maj | LK- | LK+ |  | LK+ | LK- | LK+ |  |  |  |  |
| 10-maj |  |  |  |  |  |  | LK+ | LK+ | LK+ |  |
| 11-maj | LK- |  |  |  |  |  |  |  |  |  |
| 12-maj |  | LK- |  | LK- |  | LK+ |  |  |  |  |
| 13-maj |  |  |  |  |  |  |  |  |  |  |
| 14-maj |  |  |  |  |  |  | LK+ |  |  |  |
| 15-maj |  |  |  |  |  |  |  |  |  |  |
| 16-maj |  | LK+ NS (30.88) |  | LK+ VMC (32,09) | LK- | LK+ /NS (28.97) | NS (16.4) | LK- /NS (35.05) | LK- |  |
| 17-maj |  |  |  |  |  |  |  |  |  |  |
| 18-maj | NS- | NS: (32.03) |  | NS (34.28) | NS- | NS (31.44) | NS (27.21) |  | NS- |  |
| 19-maj |  |  |  | LK+ |  |  |  | LK+ | LK- |  |
| 20-maj |  |  |  |  |  | LK- |  |  |  |  |
| 21-maj |  |  |  |  |  |  |  |  |  |  |
| 22-maj |  |  |  |  |  |  |  |  |  |  |
| 23-maj |  | LK+ |  | LK+ |  |  | LK+ |  |  |  |
| 24-maj |  |  |  |  |  |  |  |  | NS- |  |
| 25-maj |  | NS (38.55)* |  | NS (35.4)* |  |  |  |  |  |  |
| 26-maj |  |  |  |  |  |  |  |  |  |  |
| 27-maj |  |  |  |  |  |  | NS (35.92) |  |  |  |
| 28-maj |  |  |  |  |  |  |  |  |  |  |
| 29-maj |  |  |  |  |  |  |  |  |  |  |
| 30-maj |  |  |  |  |  |  |  |  |  |  |
| 31-maj |  |  |  |  |  |  |  |  |  |  |
| 01-jun |  |  |  |  |  |  | LK+ /NS (38,97)* |  |  |  |
| 02-jun |  |  |  |  |  |  |  |  |  |  |
| 03-jun |  |  |  |  |  |  |  |  |  |  |
| 04-jun |  |  |  |  |  |  |  |  |  |  |
| 05-jun |  |  |  |  |  |  |  |  |  |  |
| 06-jun |  |  |  |  |  |  |  |  |  |  |
| 07-jun |  | LK- |  |  |  |  |  |  |  |  |
| 08-jun |  |  |  |  |  |  |  |  |  |  |
| 09-jun |  |  |  |  |  |  |  |  |  |  |
| 10-jun |  | LK+ |  |  |  |  |  |  |  |  |
| 11-jun |  |  |  |  |  |  |  |  |  |  |
| 12-jun |  |  |  |  |  |  |  |  |  |  |
| 13-jun |  |  |  |  |  |  |  |  |  |  |
| 14-jun |  |  |  |  |  |  |  |  |  |  |
| 15-jun |  |  |  |  |  |  |  |  |  |  |
| 16-jun |  |  |  |  |  |  |  |  |  |  |
| 17-jun |  |  |  |  |  |  |  |  |  |  |
| 18-jun |  |  |  |  |  |  |  |  |  |  |
| 19-jun |  |  |  |  |  |  |  |  |  |  |
| 20-jun |  |  |  |  |  |  |  |  |  |  |
| 21-jun |  |  |  |  |  |  |  |  |  |  |
| 22-jun |  |  |  |  |  |  |  |  |  |  |
| 23-jun |  |  |  |  |  |  |  |  |  |  |
| 24-jun |  |  |  |  |  |  |  |  |  |  |
| 25-jun |  |  |  |  |  |  |  |  |  |  |
| 26-jun |  |  |  |  |  |  |  |  |  |  |
| 27-jun |  |  |  |  |  |  |  |  |  | LK+ |
| 28-jun |  |  |  |  |  |  |  |  |  |  |
| 29-jun |  |  |  |  |  |  |  |  |  |  |
| 30-jun |  |  |  |  |  |  |  |  |  |  |
| 01-jul |  |  |  |  |  |  |  |  |  | NS (21.48) |

**Additional File 1. Equine herpes virus 4 (EHV-4) real-time qPCR results from Laboklin and the VCM laboratory at the University of Copenhagen of the ten horses at the different dates.**

LK+ = EHV-4 PCR positive nasal swab analyzed at Laboklin. LK- = EHV-4 negative nasal swab analyzed at Laboklin. NS (Ct value) = EHV-4 positive nasal swab analyzed at the Section of Veterinary Clinical Microbiology at the University of Copenhagen (VCM) and NS- = EHV-4 negative sample analyzed at VCM. If a sample is marked with an “*” the sample only tested positive in a single reaction and not in duplicates. ^ indicates that the results are derived from a serum sample.
