# Supplementary material for: Outbreak of equine herpesvirus 4 (EHV-4) in Denmark: tracing patient zero and viral characterization
Source: BMC Vet Res. 2024 Jul 3;20:287. doi: 10.1186/s12917-024-04149-x (PMC11221098; doi:10.1186/s12917-024-04149-x)
Supplement: Supplementary file 2 — Supplementary Material 2 [file 12917_2024_4149_MOESM2_ESM.docx]

| Horse ID | Description | Reason for admission | Clinical signs related to EHV4 |
| --- | --- | --- | --- |
| Eq1 | 9 months old  Mare | Ectopic tooth | Anorexia (4 days duration) and unilateral purulent ocular discharge. |
| Eq2 | 10 months old  Gelding | Lameness and castration | History of cough, nasal discharge, and pyrexia two weeks before hospitalization. None during hospitalization. |
| Eq3 | 11 months old  Stallion | Castration | Serous unilateral ocular discharge. |
| Eq4 | 10 years old  Gelding | Colic surgery | None during hospitalization. |
| Eq5 | 14 years old  Gelding | Riding issues | Serous unilateral ocular discharge. |
| Eq6 | 11 months old  Stallion | Castration | Serous unilateral/ bilateral ocular discharge. |
| Eq7 | 1 year old  Mare | Herniorrhaphy | Mucous bilateral ocular discharge. |
| Eq8 | 2 years old  Gelding | Post castration jejunal eventration | Intermittent anorexia. |
| Eq9 | 14 years old  Gelding | Tieback surgery | None during hospitalization. |
| Eq10 | 7 years old  Gelding | Hospitalized for removal of sarcoids | History of pyrexia and lethargy one day after admission. Hospitalized after 5 days and was found EHV4 positive one day after discharge, hospitalized again after 5 days. |

**Additional File 2. Additional description, reason for admission and clinical signs of the involved horses.**
