# Supplementary material for: Outbreak of equine herpesvirus 4 (EHV-4) in Denmark: tracing patient zero and viral characterization
Source: BMC Vet Res. 2024 Jul 3;20:287. doi: 10.1186/s12917-024-04149-x (PMC11221098; doi:10.1186/s12917-024-04149-x)
Supplement: Supplementary file 3 — Supplementary Material 3 [file 12917_2024_4149_MOESM3_ESM.docx]

|  | **Eq1** | | | | **Eq2** | | | | **Eq3** | | | | **Eq4** | | | | **Eq5** | | | |
| --- | --- | --- | --- | --- | --- | --- | --- | --- | --- | --- | --- | --- | --- | --- | --- | --- | --- | --- | --- | --- |
|  | T | Lnn | ND | L/C | T | Lnn | ND | L/C | T | Lnn | ND | L/C | T | Lnn | ND | L/C | T | Lnn | ND | L/C |
|  |  |  |  |  |  |  |  |  |  |  |  |  |  |  |  |  |  |  |  |  |
| 17-04-22 |  |  |  |  |  |  |  |  |  |  |  |  | 37.9 |  |  |  |  |  |  |  |
| 18-04-22 |  |  |  |  |  |  |  |  |  |  |  |  | 37.8 |  |  |  |  |  |  |  |
| 19-04-22 |  |  |  |  |  |  |  |  |  |  |  |  | 37.8 |  |  |  |  |  |  |  |
| 20-04-22 |  |  |  |  |  |  |  |  |  |  |  |  | 37.5 |  |  |  |  |  |  |  |
| 21-04-22 |  |  |  |  |  |  |  |  |  |  |  |  | 38 |  |  |  |  |  |  |  |
| 22-04-22 |  |  |  |  |  |  |  |  |  |  |  |  | 37.7 |  |  |  |  |  |  |  |
| 23-04-22 |  |  |  |  |  |  |  |  |  |  |  |  | 37.4 |  |  |  |  |  |  |  |
| 24-04-22 |  |  |  |  |  |  |  |  | 37.8 |  |  |  | 37.5 |  |  |  |  |  |  |  |
| 25-04-22 | 38.3 |  |  |  |  |  |  |  |  |  |  |  | 38 |  |  |  |  |  |  |  |
| 26-04-22 | 38.7 |  |  |  | 38.4 |  |  | C | 38 |  |  |  | 37.6 |  |  |  | 37.5 |  |  |  |
| 27-04-22 | 37.8 |  |  |  | 38 |  |  | C | 40.2 |  |  |  | 37.3 |  |  |  | 37.8 |  |  |  |
| 28-04-22 | 38.2 |  |  |  | 38.2 |  |  | C | 40.6 |  |  |  | 37.3 |  |  |  | 37.4 |  |  |  |
| 29-04-22 | 38 |  |  |  | 38.4 |  |  |  | 38.3 |  |  |  | 38 |  |  |  | 37 |  |  |  |
| 30-04-22 | 38.1 |  |  |  | 38.4 |  |  |  | 38.6 |  |  |  | 37.2 |  |  |  | 37.3 |  |  |  |
| 01-05-22 | 37.9 |  |  |  | 38.3 |  |  |  | 38 |  |  |  | 37.2 |  |  |  | 37 |  |  |  |
| 02-05-22 | 38.1 |  |  |  | 38.2 |  |  |  | 37.7 |  |  |  | 37.1 |  |  |  | 37 |  |  |  |
| 03-05-22 | 38 |  |  |  | 38.3 |  |  |  | 37.7 |  |  |  | 38 |  |  |  | 37.5 |  |  |  |
| 04-05-22 | 37.7 |  |  |  | 38 |  |  | C | 37.9 |  |  |  | 37.6 |  |  |  | 37.6 |  |  |  |
| 05-05-22 | 38.1 |  |  |  | 37.9 |  |  |  | 37.9 |  |  |  | 37.9 |  |  |  | 37.5 |  |  |  |
| 06-05-22 | 38 |  |  |  | 38 |  |  |  |  |  |  |  | 37.7 |  |  |  | 37.5 |  |  |  |
| 07-05-22 | 37.8 |  |  | C | 38 |  |  |  |  |  |  |  | 37.6 |  |  |  | 37.6 |  |  |  |
| 08-05-22 | 37.6 |  |  |  | 38.1 |  |  |  |  |  |  |  | 37.5 |  |  |  | 37.5 |  |  |  |
| 09-05-22 | 38 |  |  |  | 37.9 |  |  |  |  |  |  |  | 37.6 |  |  |  | 37.5 |  |  |  |
| 10-05-22 | 38.1 |  |  |  | 38.1 |  |  |  |  |  |  |  | 37.6 |  |  |  | 37.1 |  |  |  |
| 11-05-22 | 38.1 |  |  |  | 38 |  |  |  |  |  |  |  | 37.5 |  |  |  | 37.1 |  |  |  |
| 12-05-22 |  |  |  |  | 37.7 |  |  |  |  |  |  |  | 37.3 |  |  |  | 37.4 |  |  |  |
| 13-05-22 | 38.2 |  |  |  | 37.9 |  |  |  |  |  |  |  | 37.4 |  |  |  | 37.5 |  |  |  |
| 14-05-22 | 38.6 |  |  |  | 37.8 |  |  |  |  |  |  |  | 37.4 |  |  |  | 37.8 |  |  |  |
| 15-05-22 | 37.7 |  |  |  | 38.1 |  |  | C |  |  |  |  | 37.5 |  |  |  | 37.4 |  |  |  |
| 16-05-22 | 38 |  |  |  | 38.2 |  |  |  |  |  |  |  | 37.8 |  |  |  | 37 |  |  |  |
| 17-05-22 | 37.7 |  |  |  | 38.7 |  |  |  |  |  |  |  | 37.3 |  |  |  | 37.1 |  |  |  |
| 18-05-22 | 38 |  |  |  | 38 |  |  |  |  |  |  |  | 37.5 |  |  |  | 37.3 |  |  |  |
| 19-05-22 | 37.7 |  |  |  | 38 |  |  |  |  |  |  |  | 37 |  |  |  | 37.3 |  |  |  |
| 20-05-22 |  |  |  |  | 38.3 |  |  |  |  |  |  |  | 37.5 |  |  |  |  |  |  |  |
| 21-05-22 |  |  |  |  | 37.8 |  |  |  |  |  |  |  | 37.2 |  |  |  |  |  |  |  |
| 22-05-22 |  |  |  |  | 37.9 |  |  |  |  |  |  |  | 37.4 |  |  |  |  |  |  |  |
| 23-05-22 |  |  |  |  |  |  |  |  |  |  |  |  | 37.8 |  |  |  |  |  |  |  |
| 24-05-22 |  |  |  |  | 38 |  |  |  |  |  |  |  | 37.3 |  |  |  |  |  |  |  |
| 25-05-22 |  |  |  |  | 38.1 |  |  |  |  |  |  |  | 37.8 |  |  |  |  |  |  |  |
| 26-05-22 |  |  |  |  | 38.1 |  |  |  |  |  |  |  | 37.4 |  |  |  |  |  |  |  |
| 27-05-22 |  |  |  |  | 37.9 |  |  |  |  |  |  |  | 37.8 |  |  |  |  |  |  |  |
| 28-05-22 |  |  |  |  | 37.9 |  |  |  |  |  |  |  | 37.4 |  |  |  |  |  |  |  |
| 29-05-22 |  |  |  |  | 38 |  |  |  |  |  |  |  | 37.4 |  |  |  |  |  |  |  |
| 30-05-22 |  |  |  |  | 37.9 |  |  |  |  |  |  |  | 37.5 |  |  |  |  |  |  |  |
| 31-05-22 |  |  |  |  | 37.6 |  |  |  |  |  |  |  | 37.6 |  |  |  |  |  |  |  |
| 01-06-22 |  |  |  |  | 37.6 |  |  |  |  |  |  |  |  |  |  |  |  |  |  |  |
| 02-06-22 |  |  |  |  | 37.7 |  |  |  |  |  |  |  |  |  |  |  |  |  |  |  |
| 03-06-22 |  |  |  |  | 37.9 |  |  |  |  |  |  |  |  |  |  |  |  |  |  |  |
| 04-06-22 |  |  |  |  |  |  |  |  |  |  |  |  |  |  |  |  |  |  |  |  |
| 05-06-22 |  |  |  |  | 37.6 |  |  |  |  |  |  |  |  |  |  |  |  |  |  |  |
| 06-06-22 |  |  |  |  | 37.3 |  |  |  |  |  |  |  |  |  |  |  |  |  |  |  |
| 07-06-22 |  |  |  |  | 38.3 |  |  |  |  |  |  |  |  |  |  |  |  |  |  |  |
| 08-06-22 |  |  |  |  | 37.6 |  |  |  |  |  |  |  |  |  |  |  |  |  |  |  |
| 09-06-22 |  |  |  |  | 38 |  |  |  |  |  |  |  |  |  |  |  |  |  |  |  |
| 10-06-22 |  |  |  |  | 38.1 |  |  |  |  |  |  |  |  |  |  |  |  |  |  |  |
| 11-06-22 |  |  |  |  | 37.8 |  |  |  |  |  |  |  |  |  |  |  |  |  |  |  |
| 12-06-22 |  |  |  |  | 37.9 |  |  |  |  |  |  |  |  |  |  |  |  |  |  |  |
| 13-06-22 |  |  |  |  | 38 |  |  |  |  |  |  |  |  |  |  |  |  |  |  |  |
| 14-06-22 |  |  |  |  | 38 |  |  |  |  |  |  |  |  |  |  |  |  |  |  |  |
| 15-06-22 |  |  |  |  | 38 |  |  |  |  |  |  |  |  |  |  |  |  |  |  |  |
| 16-06-22 |  |  |  |  | 37.9 |  |  |  |  |  |  |  |  |  |  |  |  |  |  |  |

|  | **Eq6** | | | | **Eq7** | | | | **Eq8** | | | | **Eq9** | | | |
| --- | --- | --- | --- | --- | --- | --- | --- | --- | --- | --- | --- | --- | --- | --- | --- | --- |
|  | T | Lnn | ND | L/C | T | Lnn | ND | L/C | T | Lnn | ND | L/C | T | Lnn | ND | L/C |
|  |  |  |  |  |  |  |  |  |  |  |  |  |  |  |  |  |
| 17-04-22 |  |  |  |  |  |  |  |  |  |  |  |  |  |  |  |  |
| 18-04-22 |  |  |  |  |  |  |  |  |  |  |  |  |  |  |  |  |
| 19-04-22 |  |  |  |  |  |  |  |  |  |  |  |  |  |  |  |  |
| 20-04-22 |  |  |  |  |  |  |  |  |  |  |  |  |  |  |  |  |
| 21-04-22 |  |  |  |  |  |  |  |  |  |  |  |  |  |  |  |  |
| 22-04-22 |  |  |  |  |  |  |  |  | 38.3 |  |  |  |  |  |  |  |
| 23-04-22 |  |  |  |  |  |  |  |  | 38.6 |  |  |  |  |  |  |  |
| 24-04-22 |  |  |  |  |  |  |  |  | 37.5 |  |  |  |  |  |  |  |
| 25-04-22 | 37.6 |  |  |  |  |  |  |  | 38 |  |  |  |  |  |  |  |
| 26-04-22 | 38 |  |  |  |  |  |  |  | 37.8 |  |  |  |  |  |  |  |
| 27-04-22 | 38.1 |  |  |  |  |  |  |  | 38.2 |  |  |  |  |  |  |  |
| 28-04-22 | 38.1 |  |  |  |  |  |  |  | 38.1 |  |  |  |  |  |  |  |
| 29-04-22 | 37.9 |  |  |  |  |  |  |  | 38.3 |  |  |  |  |  |  |  |
| 30-04-22 | 37.4 |  |  |  |  |  |  |  | 37.9 |  |  |  |  |  |  |  |
| 01-05-22 | 37.8 |  |  |  |  |  |  |  | 39.3 |  |  |  | 37.6 |  |  |  |
| 02-05-22 | 37.3 |  |  |  |  |  |  |  | 40 |  |  |  | 37.6 |  |  |  |
| 03-05-22 | 38.4 |  |  |  | 39 |  |  |  | 39.7 |  |  |  | 38.1 |  |  |  |
| 04-05-22 | 38.4 |  |  | C | 40.2 |  |  |  | 39.6 |  |  |  | 37.9 |  |  |  |
| 05-05-22 | 38.4 |  |  |  | 38.4 |  |  |  | 39.2 |  |  |  | 37.9 |  |  |  |
| 06-05-22 | 38 |  |  |  | 38.7 |  |  |  | 38 |  |  |  | 37.6 |  |  |  |
| 07-05-22 | 37.8 |  |  |  | 38.7 |  |  |  | 38 |  |  |  | 37.9 |  |  |  |
| 08-05-22 | 37.7 |  |  |  | 37.5 |  |  |  | 37.8 |  |  |  | 38 |  |  |  |
| 09-05-22 | 37.6 |  |  |  |  |  |  |  | 37.1 |  |  |  | 37.6 |  |  |  |
| 10-05-22 | 37.8 |  |  |  | 38.1 |  |  |  | 37.5 |  |  |  | 37.3 |  |  |  |
| 11-05-22 | 37.8 |  |  |  | 37.9 |  |  |  | 37.5 |  |  |  | 37.6 |  |  |  |
| 12-05-22 | 38.2 |  |  |  | 37.7 |  |  |  |  |  |  |  |  |  |  |  |
| 13-05-22 | 38 |  |  |  |  |  |  |  | 37.6 |  |  |  | 37.6 |  |  |  |
| 14-05-22 | 38 |  |  |  | 38.1 |  |  |  | 37.9 |  |  |  | 37.7 |  |  |  |
| 15-05-22 | 37.7 |  |  |  |  |  |  |  | 37.5 |  |  |  | 37.8 |  |  |  |
| 16-05-22 |  |  |  |  |  |  |  |  |  |  |  |  |  |  |  |  |
| 17-05-22 | 37.7 |  |  |  |  |  |  |  | 38.1 |  |  |  | 37.5 |  |  |  |
| 18-05-22 | 37.3 |  |  |  |  |  |  |  | 37.7 |  |  |  | 37.8 |  |  |  |
| 19-05-22 |  |  |  |  | 37.9 |  |  |  | 37.8 |  |  |  | 37.9 |  |  |  |
| 20-05-22 |  |  |  |  | 37.7 |  |  |  | 37.8 |  |  |  | 37.8 |  |  |  |
| 21-05-22 |  |  |  |  |  |  |  |  |  |  |  |  | 37.7 |  |  |  |
| 22-05-22 |  |  |  |  |  |  |  |  |  |  |  |  | 37.8 |  |  |  |
| 23-05-22 |  |  |  |  |  |  |  |  |  |  |  |  |  |  |  |  |
| 24-05-22 |  |  |  |  |  |  |  |  | 37.7 |  |  |  | 37.8 |  |  |  |
| 25-05-22 |  |  |  |  |  |  |  |  | 37.3 |  |  |  |  |  |  |  |
| 26-05-22 |  |  |  |  | 37.8 |  |  |  | 37.7 |  |  |  |  |  |  |  |
| 27-05-22 |  |  |  |  | 37.3 |  |  |  | 37.9 |  |  |  |  |  |  |  |
| 28-05-22 |  |  |  |  |  |  |  |  |  |  |  |  |  |  |  |  |
| 29-05-22 |  |  |  |  |  |  |  |  |  |  |  |  |  |  |  |  |
| 30-05-22 |  |  |  |  | 37.9 |  |  |  |  |  |  |  |  |  |  |  |
| 31-05-22 |  |  |  |  | 37.6 |  |  |  |  |  |  |  |  |  |  |  |
| 01-06-22 |  |  |  |  | 37.9 |  |  |  | 37.2 |  |  |  |  |  |  |  |
| 02-06-22 |  |  |  |  | 38.1 |  |  |  |  |  |  |  |  |  |  |  |
| 03-06-22 |  |  |  |  |  |  |  |  |  |  |  |  |  |  |  |  |
| 04-06-22 |  |  |  |  |  |  |  |  |  |  |  |  |  |  |  |  |
| 05-06-22 |  |  |  |  |  |  |  |  |  |  |  |  |  |  |  |  |
| 06-06-22 |  |  |  |  | 37.7 |  |  |  |  |  |  |  |  |  |  |  |
| 07-06-22 |  |  |  |  | 38 |  |  |  |  |  |  |  |  |  |  |  |
| 08-06-22 |  |  |  |  | 38.1 |  |  |  |  |  |  |  |  |  |  |  |

| **Eq10** | | | | |
| --- | --- | --- | --- | --- |
|  | T | Lnn | ND | L/C |
| 15-06-22 | 37.7 |  |  |  |
| 16-06-22 | 37.8 |  |  |  |
| 17-06-22 | 37.5 |  |  |  |
| 18-06-22 |  |  |  |  |
| 19-06-22 |  |  |  |  |
| 20-06-22 |  |  |  |  |
| 21-06-22 |  |  |  |  |
| 22-06-22 |  |  |  |  |
| 23-06-22 |  |  |  |  |
| 24-06-22 | 38.5 |  |  | C |
| 25-06-22 | 39.8 |  |  |  |
| 26-06-22 | 38.9 |  |  |  |
| 27-06-22 | 39.2 |  |  |  |
| 28-06-22 | 38.1 |  |  |  |
| 29-06-22 | 37.6 |  |  |  |
| 30-06-22 | 37.6 |  |  |  |
| 01-07-22 | 37.7 |  |  |  |
| 02-07-22 | 37.8 |  |  |  |
| 03-07-22 | 37.6 |  |  |  |
| 04-07-22 | 37.5 |  |  |  |

**Additional File 3. Clinical registrations of pyrexia, mandibular lymphadenopathy, nasal discharge, coughing and increased lung sound for each of the ten horses**

T = rectal temperature, Lnn = lymphonoduli mandibularis, NS = nasal discharge, L/C = lung sound upon auscultation and coughing.

Heat map presents the results of four different clinical registrations for each horse at specific dates. A Green color in the column “T” indicates a normal temperature ≤ 37.9°C, yellow indicates subfebrile temperature between 38.0-38.4°C and red indicates a pyrexia ≥38.5. The grey scaled temperatures indicates that the specific horse on the specific days where treated with antipyretic medicine. Green colurs in the column “Lnn” indicates a “normal” size of the lymphnodes, yellow indicates “unilateral lymphadenopathy” and red indicates “bilateral lymphadenopathy”. Green colour in the column “ND” indicates no nasal discharge, yellow indicates unilateral nasal discharge and red indicates bilateral nasal discharge. Green colour in the column “L” indicates normal lung sounds, yellow indicates increased lung sounds and red indicates coughing.
