# Supplementary material for: Outbreak of equine herpesvirus 4 (EHV-4) in Denmark: tracing patient zero and viral characterization
Source: BMC Vet Res. 2024 Jul 3;20:287. doi: 10.1186/s12917-024-04149-x (PMC11221098; doi:10.1186/s12917-024-04149-x)
Supplement: Supplementary file 4 — Supplementary Material 4 [file 12917_2024_4149_MOESM4_ESM.docx]

|  | Eq1 | Eq2 | Eq3 | Eq4 | Eq5 | Eq6 | Eq7 | Eq8 | Eq9 | Eq10 |
| --- | --- | --- | --- | --- | --- | --- | --- | --- | --- | --- |
| 17.04.22 |  |  |  | S |  |  |  |  |  |  |
| 22.04.22 |  |  |  |  |  |  |  | S |  |  |
| 24.04.22 |  |  | S |  |  |  |  |  |  |  |
| 25.04.22 | S | S |  |  |  | S |  |  |  |  |
| 27.04.22 | LK | LK | LK |  |  |  |  |  |  |  |
| 28.04.22 |  |  |  |  |  |  | S |  |  |  |
| 01.05.22 |  |  |  |  |  |  |  |  | S |  |
| 02.05.22 | LK | LK | LK | LK | LK | LK |  | S + LK | LK |  |
| 03.05.22 |  |  |  |  |  |  | LK |  |  |  |
| 05.05.22 | NS + S | NS + S | NS + S | NS + S | NS + S | NS + S |  | NS + LK |  |  |
| 07.05.22 |  |  |  |  |  |  |  | NS |  |  |
| 08.05.22 |  |  |  |  |  |  | NS |  |  |  |
| 09.05.22 | LK | LK |  | LK | LK | LK |  |  |  |  |
| 10.05.22 |  |  |  |  |  |  | LK | LK | LK |  |
| 11.05.22 | LK |  |  |  |  |  |  |  |  |  |
| 12.05.22 |  | LK |  | LK |  | LK |  |  |  |  |
| 14.05.22 |  |  |  |  |  |  | LK |  |  |  |
| 16.05.22 |  | NS + LK |  | NS + LK | NS + S + LK | NS + LK | NS | NS + LK | LK |  |
| 18.05.22 | NS + S | NS + S |  | NS + S | NS + S | NS + S | NS + S |  | NS + S |  |
| 19.05.22 |  |  |  | LK |  |  |  | LK | LK |  |
| 20.05.22 |  |  |  |  |  | LK |  |  |  |  |
| 23.05.22 |  | LK |  | LK |  |  | LK |  |  |  |
| 24.05.22 |  | S |  |  |  |  |  |  | NS + S |  |
| 25.05.22 |  | NS |  | NS + S |  |  |  |  |  |  |
| 27.05.22 |  |  |  |  |  |  | NS |  |  |  |
| 01.06.22 |  |  |  |  |  |  | NS + LK |  |  |  |
| 07.06.22 |  | LK |  |  |  |  |  |  |  |  |
| 10.06.22 |  | LK |  |  |  |  |  |  |  |  |
| 15.06.22 |  |  |  |  |  |  |  |  |  | S |
| 24.06.22 |  |  |  |  |  |  |  |  |  | S |
| 01.07.22 |  |  |  |  |  |  |  |  |  | NS + S |
| 28.07.22 |  |  |  |  |  |  | S |  |  |  |

**Additional File 4. Sample list over the included samples in the study for each of the ten horses (Eq1-Eq10) at different dates**

Eq = equine, NS = nasal swabs analyzed at the Section of Veterinary Clinical Microbiology at the University of Copenhagen (VCM) for EHV-4, LK = nasal swabs analyzed at Laboklin for EHV-4, S =a serum samples analyzed for EHV-4 antibodies at VCM.
