# Supplementary material for: Outbreak of equine herpesvirus 4 (EHV-4) in Denmark: tracing patient zero and viral characterization
Source: BMC Vet Res. 2024 Jul 3;20:287. doi: 10.1186/s12917-024-04149-x (PMC11221098; doi:10.1186/s12917-024-04149-x)
Supplement: Supplementary file 5 — Supplementary Material 5 [file 12917_2024_4149_MOESM5_ESM.docx]

| ORF30 PCR primers: | | |
| --- | --- | --- |
| Primer name | | Sequence (5’- 3’) |
| ORF30-1-F | | AATCTCGAGTCAGCTTTGATGGGGAACTG |
| ORF30-1-R | | AGAACTGCCCAGTGTGAAGG |
| ORF30-2-F | | ACCCCCTTCATGAGCAT |
| ORF30-2-R | | GGAGGGCTGTTTAAGGTCTG |
| ORF30-3-F | | ATACAATACTCTCCTATTAC |
| ORF30-3-R | | ATTGCGGCCGCATGGCGGCGCACGAACAGGA |
| ORF30-4-F | | AGCAAACCGCGACGGGTCGT |
| ORF30-4-R | | ATTGCGGCCGCATGGCGGCGCACGAACAGGA |
| PCR Mix: | | |
| Per sample:  14.25 𝜇L DNase/RNase free water  2.5 𝜇L 10x Accuprime PCR buffer I  0.25 𝜇L Accuprime Taq High Fidelity  0.5 𝜇L MgSO4  1.25 𝜇L Primer-F (20𝜇M)  1.25 𝜇L Primer-R (20 𝜇M) | | |
| PCR programs: | | |
| ORF-30-1 | | |
| Hold: | 94°C – 2 min | |
| Cycling: | 40x (94°C – 30 sec, 60°C – 60 sec, 68°C – 180 sec) | |
| Hold: | 68°C – 10 min | |
| Hold: | 4°C, pause | |
|  | | |
| ORF-30-2 | | |
| Hold: | 94°C – 2 min | |
| Cycling: | 40x (94°C – 30 sec, 53°C – 90 sec, 68°C – 180 sec) | |
| Hold: | 68°C – 10 min | |
| Hold: | 4°C, pause | |
|  | | |
| ORF-30-3 | | |
| Hold: | 94°C – 2 min | |
| Cycling: | 40x (94°C – sec, 50°C – 120 sec, 68°C – 90 sec) | |
| Hold: | 68°C – 10 min | |
| Hold: | 4°C, pause | |
|  | | |
| ORF-30-4 | | |
| Hold: | 94°C – 2 min | |
| Cycling: | 40x (94°C – 30 sec, 60°C – 60 sec, 68°C – 90 sec) | |
| Hold: | 68°C – 10 min | |
| Hold: | 4°C, pause | |

**Additional File 5. Details of the PCR amplification of open reading frame 30 (ORF30)**
